# Double conditional human embryonic kidney cell line based on FLP and ΦC31 mediated transgene integration

**DOI:** 10.1186/1756-0500-4-420

**Published:** 2011-10-18

**Authors:** Christoph Waldner, Olga Rempel, Fabian Schütte, Mert Yanik, Natalie Solomentsew, Gerhart U Ryffel

**Affiliations:** 1 Institut für Zellbiologie (Tumorforschung), Universität Duisburg-Essen, Hufelandstr. 55, D-45122 Essen, Germany

## Abstract

**Background:**

FLP recombinase mediated integration into a pre-integrated FRT site is routinely used to generate highly reproducible stable transgenic cell lines. In this study, we broaden the system of site specific integration by introducing ΦC31 integrase mediated integration into attP sites.

**Results:**

We generated a HEK293 host cell line with a single copy FRT as well as an attP site allowing site specific integration of two distinct transgenes. To achieve conditional control, we used the tetracycline and Shld1 inducible systems. By introducing fluorescent reporters we show that integration and induction of two transgenes are completely independent. We applied this new technique to investigate the effect of HNF4α on proliferation of HEK293 cells by introducing HNF4α into each integration site. We obtained in two independent cell lines highly reproducible results that prove the usefulness of this novel HEK-attP/FRT cell line.

**Conclusions:**

In this study we have established and applied a HEK-attP/FRT cell line that allows site specific integration of two conditional transgenes using the FLP recombinase as well as the ΦC31 integrase.

## Background

Stable integration of inducible transgenes is widely used to analyze gene function in mammalian cells. To obtain highly reproducible results from different cell lines, site specific integration of one single transgene copy is essential. Integration into a pre-determined genomic locus can be achieved by FLP recombinase mediated integration [1] and the tetracycline inducible expression system [2] is most commonly used for conditional transgene activation. The Flp-In T-REx™ system (Invitrogen) uses a genomic FRT site for integration of any gene-of-interest (GOI) by FLP recombinase and is based on the Tet-repressor (TetR) that inhibits via two tetracycline operator (tetO) sequences the CMV promoter of the GOI. This system has successfully been used to investigate factors that control cell proliferation [3,4] and to reproducibly identify target genes of transcription factors [5]. Recently, this system was further improved by reducing the background expression of the GOI, when no inducer is added [6].

For many experiments the independent conditional expression of two distinct transgenes would be most desirable. Thus, an additional and independent conditional system is required. Recently, another inducible system was developed that allows conditional protein degradation [7]. The human FK506- and rapamycin-binding protein (FKBP12) is rapidly and constitutively degraded in mammalian cells. Protein fusion of its destabilizing domain (DD) transfers the instability to any protein-of-interest. Addition of the synthetic ligand Shld1 that binds to the destabilizing domain protects the fusion protein from rapid degradation and thus enhances abundance of the protein-of-interest.

An enzyme that acts completely independent of FLP and catalyses specific integration at high efficiency is the serine integrase derived from the phage ΦC31. This ΦC31 integrase mediates site-specific recombination between two DNA sequences, the phage attachment site, attP, and the bacterial attachment site, attB [8]. Importantly, recombining the attP and attB sites generates two hybrid sites, attL and attR, which cannot be recognized by the integrase and thus in contrast to the FLP recombinase, the reaction is unidirectional and much more efficient. In several mammalian cell lines it has been shown that a plasmid containing the attB sequences integrates with different efficiency into pseudo attP sites of the genomic DNA [9]. There are about 100 distinct integrations sites that show different frequency of integration [10]. Sequencing of these pseudo attP sites after integration showed that these recombinations were not completely precise at the sequence level, differing slightly at the integration junction. In contrast the integration into wild-type attP sites inserted into the mammalian genome was invariable precise resulting in the expected recombination event at the sequence level. This property is most valuable to design strategies to define attP site specific integrations. Comparing in the human cell line HEK293 the integration frequency between the pseudo attP sites and a wild-type attP site, about 15% of the integrations occurred at the introduced wild-type attP site. In contrast, the most frequent pseudo attP site (phiA) was used at 5% suggesting the wild-type attP site is preferred [9]. To prevent unwanted integration at pseudo attP sites a selection is needed for specific integration at a pre-existing attP docking site using corresponding markers. This can best be achieved by activation of an antibiotic resistance gene upon proper recombination [9]. Significantly, the fusion of a nuclear localization signal (NLS) to the ΦC31 integrase has shown an eightfold increase in the integrase activity [11] suggesting that this is an important modification to get optimal activity in vertebrate cells.

In this study, we combine the Shld1 inducible system with ΦC31 integrase mediated specific integration in the background of the tetracycline inducible FLP mediated system to have two completely independent ways of transgene activation in one and the same cell. We apply this technique to introduce the cell specific transcription factor HNF4α into a human embryonic kidney (HEK293) cell line and investigate its effect on cell proliferation.

## Results

### Design of the integration systems

**Figure 1:**
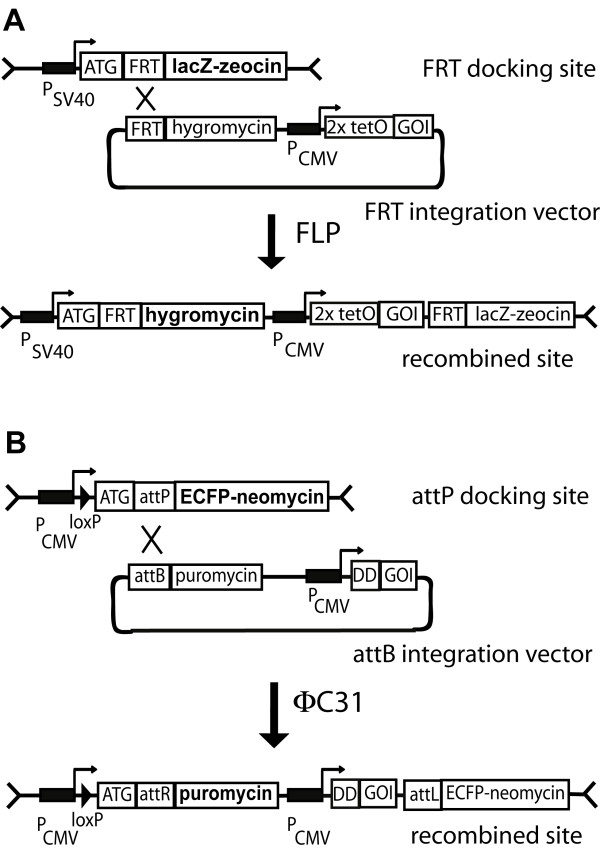
**Scheme of the two independent integration systems used**. **A**: FRT docking site is used as in the Flp-In T-Rex™ system (Invitrogen). The FRT sequence is placed downstream and in frame of the translational initiation codon ATG from the lacZ-zeocin fusion protein. After FLP mediated recombination with the FRT sequence of the integration vector, lacZ-zeocin expression is turned off and hygromycin resistance is activated. The GOI (gene-of-interest) is placed downstream of the hygromycin resistance cassette and controlled by the tetracyclin inducible CMV promoter containing the two tetracycline operators (2xtetO). **B**: attP docking site for ΦC31 integrase mediated integration. The attP sequence is placed downstream and in frame of the translational initiation codon ATG from the ECFP-Neo fusion protein. After ΦC31 mediated recombination with the attB sequence of the integration vector, ECFP-Neo expression is turned off and puromycin resistance is activated. The GOI is placed downstream of the puromycin resistance cassette and controlled by the CMV promoter. The N-terminal destabilizing domain (DD) is linked to the GOI to allow regulation by Shld1.

To allow site specific integration of two different transgenes we used two independent systems. The first is based on FLP recombinase mediated recombination of FRT sites (Figure 1A). By using hygromycin resistance, recombined cell lines can be selected. Moreover, loss of lacZ reporter activity monitors specific recombination with the FRT integration vector. Conditional transcriptional control of the transgene is achieved by applying the tetracycline inducible system. This system is well established and commercially available (Flp-In T-Rex™, Invitrogen). As a second and independent system we developed ΦC31 integrase mediated recombination of attP and attB sites (Figure 1B). To allow site directed integration of any gene-of-interest (GOI) using the ΦC31 integrase, we designed the docking construct pDOCKING-Neo containing the attP recognition site. This site is linked in frame to an ECFP-neomycin resistance fusion protein as selection marker. As the attP site is placed downstream of the start codon for ECFP-Neo, this marker will be inactivated upon specific recombination with the corresponding attB sequence. The incoming attB integration vector contains the attB site fused to a promoter- and ATG-less puromycin resistance gene, which is thus activated upon specific recombination with the attP sequence of the docking site. The GOI is placed downstream of the puromycin resistance sequence. In all experiments we used the CMV promoter and fused the GOI to the L106P mutant of the human FKBP12 protein (destabilizing domain, DD) to regulate the expression on the level of protein stability by Shld1 [7].

### Generation of double docking HEK293 cell lines

The commercially available Flp-In T-Rex™ HEK293 cells contain a stably integrated single copy FRT docking site for FLP mediated integration. Using this cell line we introduced in addition for ΦC31 mediated transgene integration the newly generated attP docking site that encodes an ECFP-Neo fusion gene. After selection with G418 we selected 24 single clones with blue fluorescence. To determine the number of integrated transgene copies we performed real-time PCR on genomic DNA and selected the three double docking cell lines 12, 16, and 19 with apparent single copy integration of the docking site for further experiments.

### Independent integration of inducible fluorescent proteins into different double docking HEK293 cell lines

To test whether independent integration as well as independent conditional activation of two different GOI can be achieved in the double docking cell lines we generated two different integration vectors. The FRT integration vector pcDNA5/FRT/TO-DsRed contains the red fluorescent protein DsRed as GOI (Figure 1A), whereas the attP integration vector pINT-PuroDDEYFP contains as GOI the yellow fluorescent protein EYFP linked to the destabilizing domain (DD in Figure 1B). In a first round of transfection, the DsRed gene was introduced using the FLP recombinase. The obtained cell lines from each clone were used for a second round of transfection using the EYFP containing attP integration vector and the ΦC31 integrase. The resulting cell lines were resistant to hygromycin and puromycin and have thus potentially integrated the two transgenes.

**Figure 2:**
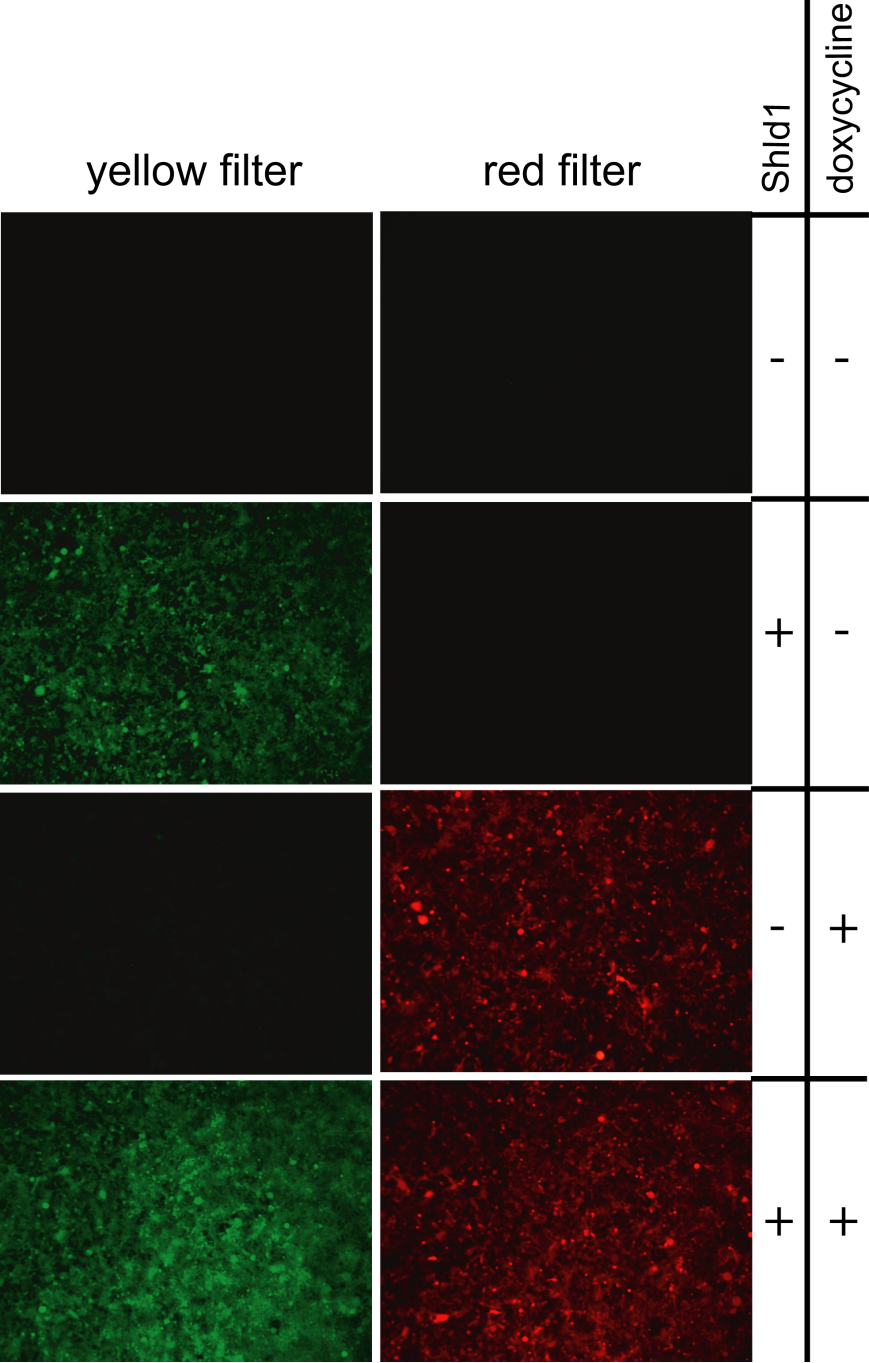
**Independent integration and induction of fluorescent reporters**. Cells of the cell line 19-3-1 have the Shld1 inducible DD-EYFP protein integrated into the attP docking site and the doxycycline inducible DsRed protein integrated into the FRT docking site. Cells were cultured in six well plates and incubated with 1 μg/ml doxycycline or 1 μM Shld1 as indicated. After 24 h fluorescence was observed and documented using a Leica MZ-FL III with appropriate filter sets as indicated.

**Figure 3:**
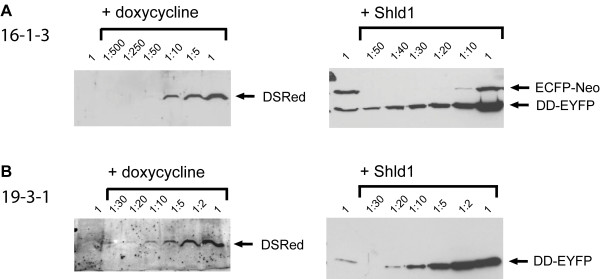
**Quantification of reporter activation by western blot**. Lysates of cell line 16-1-3 (**A**) or 19-3-1 (**B**) were probed by western blots using an anti-DsRed antibody (left panels) or an anti-GFP antibody (right panels). Cell lysates were diluted as indicated. Note that the GFP antibody recognizes EYFP as well as ECFP. 1 μg/ml doxycycline or 1 μM Shld1 was added as indicated.

To test whether the two transgenes were present and conditionally active, we treated all cell lines with doxycycline, a relatively stable tetracycline derivative, or Shld1 and monitored red or yellow fluorescence (Figure 2). In all cell lines derived from double docking cell lines 12, 16, and 19, red fluorescence was specifically induced by doxycycline and yellow fluorescence by Shld1 in more than 90% of the cells. As an example, a cell line derived from double docking cell line 19 is shown in Figure 2. Clearly, there was no cross-reaction between the two conditional systems, and both genes can be induced in parallel. Next, we quantified the level of induction by western blot analyses. Doxycycline treatment caused an about 50-fold increased DsRed expression in the cell lines 16-1-3 (Figure 3A) and 12-1-1 (not shown), and about 20-fold induction in 19-3-1 (figure 3B). When Shld1 was given to the cells, the DD-EYFP protein was induced about 30-fold in 16-1-3 (Figure 3A), 20-fold in 12-1-1 (not shown), and 20-fold in 19-3-1 (Figure 3B). Of note, in the cell lines 16-1-3 (Figure 3A) and 12-1-1 (not shown) the ECFP-Neo fusion protein was still expressed. We assume that in these cell lines there is at least one un-recombined copy of the attP docking site present, although the transgene was integrated. In contrast, the cell line 19-3-1 lacks ECFP-Neo expression arguing for recombination at a unique attP docking site. Thus, the cell line 19, we refer to as HEK-attP/FRT cell line, is best suited to achieve stable integration of two distinct transgenes with independent conditional activation of each transgene.

### Independent integration of two inducible HNF4α proteins

**Figure 4:**
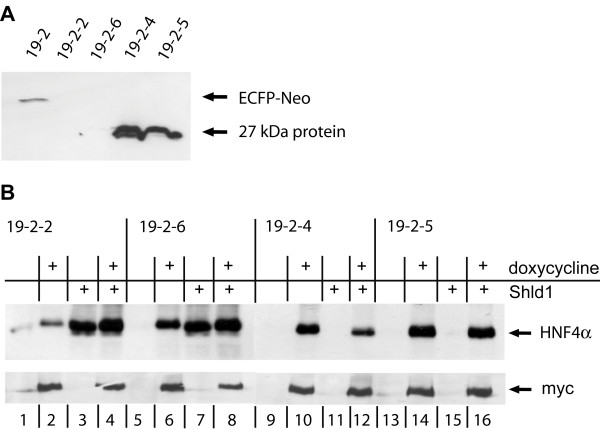
**Independent integration of two inducible HNF4α proteins**. **A**: Western blot analysis was performed on the 19-2 parent cell line containing the myc tagged HNF4α2 coding region in the FRT site and on the four ΦC31 mediated descendent cell lines 19-2-2, 19-2-6, 19-2-4, and 19-2-5. The anti-GFP antibody recognizes ECFP. **B**: Western blot analysis of the cell lines 19-2-2, 19-2-6, 19-2-4, and 19-2-5 using an anti-HNF4α or anti-myc antibody. The cells were cultured in six well plates, incubated with 1 μg/ml doxycycline and/or 1 μM Shld1 as indicated and protein extracts prepared after 24 h. As the HNF4α transgene introduced into the FRT docking site has a myc-tag, it can be detected specifically with the anti-myc antibody. The anti-HNF4α antibody detects the myc-tagged as well as the DD-tagged HNF4α proteins that differ slightly in size (compare e.g. lanes 2 and 3).

To verify that this double conditional system can also be used to express genes interfering with cell cycle progression we introduced the transcription factor HNF4α splice variant 2. (HNF4α2) that has been shown to inhibit HEK293 cell multiplication [3,4] into each docking site. In a first round of transfection, the doxycycline inducible HNF4α2 sequence was introduced into the FRT site of the HEK-attP/FRT cells using the FLP recombinase. Site specific integration was verified by negative lacZ staining in three independent cell lines. One cell line 19-2 was used for a second round of transfection to integrate Shld1 inducible HNF4α2 into the attP docking site using ΦC31 integrase. The resulting four cell lines (19-2-2, 19-2-6, 19-2-4 and 19-2-5) were resistant to hygromycin and puromycin and have thus potentially integrated the two transgenes. To verify site specific integration into the attP docking site we screened for loss of ECFP-Neo expression, which is inactivated upon recombination (compare Figure 1B). This loss of ECFP-Neo expression was validated by a western blot using a monoclonal anti-GFP antibody also detecting ECFP (Figure 4A). The parent cell line 19-2 shows expression of the 61 kDa ECFP-Neo fusion protein, whereas no expression was detectable in the cell lines 19-2-2 and 19-2-6 arguing for specific integration of the HNF4α transgene in these cells. In contrast, the cell lines 19-2-4 and 19-2-5 expressed an about 27 kDa protein recognized by the anti-GFP antibody. In these two later cell lines the attP docking site was possibly rearranged by illegitimate recombination resulting in a truncated ECFP-Neo protein. We then tested by western blot analyses whether HNF4α could be induced in these cell lines by doxycycline or Shld1. As the doxycycline inducible HNF4α transgene contains a myc tag, we could differentiate between the doxycycline and Shld1 inducible proteins. In all four cell lines the myc-tagged HNF4α transgene could be induced specifically by doxycycline to a most similar level (Figure 4B, lower panel). However, when treated with Shld1 the cell lines 19-2-2 and 19-2-6 showed activation of HNF4α, whereas the cell lines 19-2-4 and 19-2-5 lacked HNF4α expression despite of puromycin resistance indicating ΦC31 mediated integration of the transgene. The ability to induce HNF4α by Shld1 in the cell lines 19-2-2 and 19-2-4 correlates with the loss of ECFP-Neo expression indicating specific integration in these two cell lines. In contrast, the cell lines 19-2-4 and 19-2-5 still expressing a truncated ECFP-Neo protein were not inducible by Shld1.

**Figure 5:**
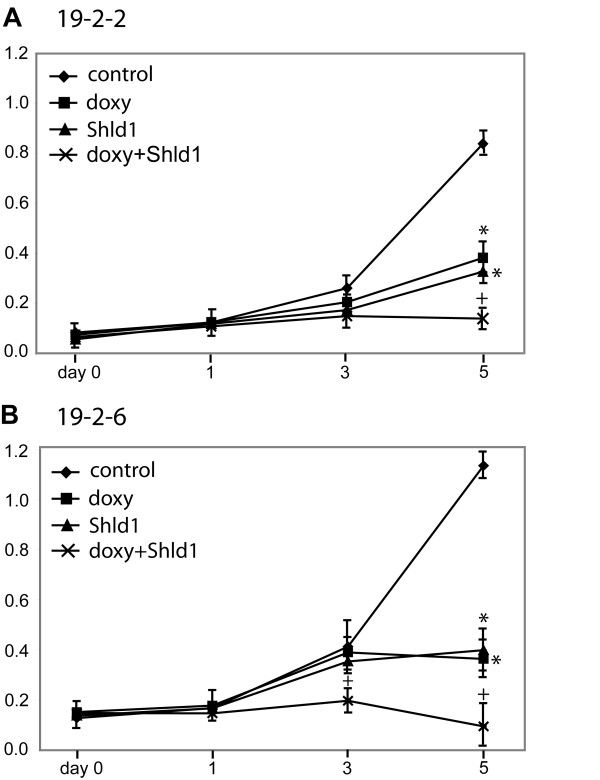
**Effect of HNF4α on cell proliferation**. MTS proliferation assay was performed over a time span of five days in cell line 19-2-2 (**A**) or cell line 19-2-6 (**B**). Cells were treated with 1 μg/ml doxycycline and/or 1 μM Shld1 at day 0 as indicated and the metabolic activity was determined at the given time points. The medium was not changed during the whole period. The assay was performed in 96 well plates with n = 3 measurements. Using the student's t-test p-values were calculated for doxycycline or Shld1 treatment compared to control: *p < 0.01. Similarly, p-values comparing double treated samples (doxycycline and Shld1) with doxycycline treated samples were computed: ^+^p < 0.01. The results were confirmed in an additional independent experiment for each cell line. We did not observe any effects of doxycycline or Shld1 on cell proliferation of HEK293 cells without the HNF4α transgene.

To address the effect of both HNF4α transgenes on cell cycle progression we treated the cell lines 19-2-2 and 19-2-6 with doxycycline and/or Shld1 and measured the cell number over five days by the MTS assay. The cell line 19-2-2 showed an about 10-fold increase of cell mass until day 5 (Figure 5A). However, when doxycycline or Shld1 was given cell multiplication was significantly reduced by 5-fold or 6-fold, respectively. Significantly, administration of doxycycline and Shld1 in parallel retained the cell number at about the starting levels pointing to an additive effect of the two independent HNF4α transgenes. MTS assay of the 19-2-6 cell line (Figure 5B) showed most similar results with a significant reduction of cell multiplication by either doxycycline or Shld1 treatment and a lack of cell multiplication, if doxycyline and Shld1 were applied simultaneously.

In summary, the two HNF4α transgenes could be independently activated by doxycycline or Shld1 and the observed effect on cell multiplication was highly reproducible between the two distinct sites of integration in two individual cell lines. Moreover, the effect of HNF4α appears to be concentration dependent, as activation of the second transgene further delays cell multiplication.

## Discussion

In this study we created the double conditional cell line HEK-attP/FRT to independently control the expression of two stably integrated transgenes. While stable cell lines are superior to transient transfectants to investigate long term effects, there is considerable variation between different clones as the site of integration and number of transgene copies cannot be predicted using standard transfection methods. This variation becomes even more problematic, when two potentially interacting transgenes are studied. This problem has now been overcome by using in parallel FLP and ΦC31 integrase mediated integration into the corresponding pre-integrated recognition sites.

When searching for the optimal HEK-attP/FRT docking cell line, it turned out that two of three host cell lines still expressed the marker for the native attP docking site after integration of the fluorescent proteins. As the cells were resistant to puromycin and the fluorescent proteins could be properly induced, we conclude that there is least one additional un-recombined copy of the attP docking site present in these cells. However, the finally selected HEK-attP/FRT cell line appears useful for single copy integration, as it allows recombination at a unique site resulting in loss of the attP marker gene expression (19-3-1 in Figure 3A). All experiments confirm that induction by doxycycline or Shld1 is completely independent (Figure 2, 3 and 4). This could be expected, as gene activation by doxycycline (or tetracycline, tet-On system) acts on the transcriptional level [2], while Shld1 affects protein stability [7]. To have both, transcriptional and post-translational control, in one cell makes this system most versatile. The fold induction was comparable between the two systems (Figure 4B), although we cannot exclude that the myc tagged HNF4α protein and the DD-HNF4α fusion protein, in the presence of Shld1, have distinct stabilities. While in theory ΦC31 integrates more efficiently than FLP [8], we did not obtain more clones by ΦC31 mediated integration than by FLP in our experiments.

Integrating HNF4α into the HEK-attP/FRT cell line it appeared necessary to screen for correct integration by loss of ECFP-Neo expression (Figure 4). Illegitimate recombination or integration at pseudo attP sites can probably not completely be avoided by puromycin selection, but the loss of ECFP-Neo expression (cell lines 19-2-2 and 19-2-6) correlated perfectly with proper Shld1 inducibility in these two cell lines. The occurrence of unspecific integration was comparable to unspecific recombination events observed using FLP mediated integration that results in a high proportion of lacZ positive cells (own observations). The fact that doxycycline or Shld1 induced HNF4α has nearly the same quantitative effect on cell proliferation in two independent cell lines (Figure 5) implies that the two distinct HNF4α fusion proteins integrated at two distinct loci have most similar physiological properties. Of note, there was also an additive effect, if the two copies were activated simultaneously. This underlines the usefulness of the double conditional cell line HEK-attP/FRT to study the function of two potentially synergistic factors. Future applications for this novel cell line will also include the introduction of two different proteins with opposing functions allowing the study of any induced downstream signaling. Recently, another technique to introduce two different transgenes into two distinct sites was established using recombinase mediated cassette exchanged (RMCE) [12], but no conditional control of transgene expression was applied. While the system of multiplexing RMCE has the advantage of introducing two transgenes in one step, no specific selection markers for integration into each site were used. The integration of the transgenes in two stages as in our system is time consuming. One possibility to speed up this process could be to transfect the host cells in parallel with FLP and ΦC31 integrase and the corresponding integration vectors in a single transfection. However, we did not test this ambitious option. During preparation of our manuscript a multi-integrase system involving even five distinct recombination sites has been described [13]. However, our system allows in addition the conditional expression of two independent genes integrated at well defined genomic positions.

## Conclusions

In this study we have established and applied a HEK-attP/FRT cell line that allows site specific integration of two distinct transgenes using the FLP recombinase as well as the ΦC31 integrase. Moreover, this approach implicates two specific conditional systems to control transgene expression.

## Methods

### Cell culture and transfection

All cell lines were grown in Dulbecco's modified Eagle's medium (DMEM, Gibco-BRL) supplemented with 10% heat inactivated fetal calf serum (FCS), penicillin/streptomycin (100 U/ml) and 2 mM glutamine at 37°C under 8% CO_2 _atmosphere and a relative humidity of 95%. The cell line Flp-In T-Rex™ 293 (Invitrogen) was cultured in DMEM supplemented with 15 μg/ml blasticidin and 100 μg/ml zeocin (InvivoGen). The HEK-attP/FRT cell line was kept in DMEM supplemented with blasticidin, zeocin, and 500 μg/ml G418 (InvivoGen). To select stable cell lines derived from the host cell line by FLP mediated integration into the FRT docking site, zeocin was substituted with 50 μg/ml hygromycin B (Invitrogen). After ΦC31 integrase mediated integration into the attP docking site, G418 was replaced by 1 μg/ml puromycin (InvivoGen). Transfections were carried out using LipofectAMINE™ (Invitrogen) or FuGENE™ (Roche).

### Generation and induction of stable cell lines

For integration into the FRT docking site, cells were co-transfected with the FLP expression vector pCSFLPe [14] and the corresponding integration vector at a ratio of 9 to 1. As the transgenes are integrated at the same chromosomal site, cell lines were prepared by pooling individual colonies. Successful integration was verified by staining the cells for β-galactosidase activity. Cell lines with less than 5% blue cells were used for further experiments. For integration into the attP docking site, cells were co-transfected with the ΦC31 integrase expression vector pchactC31hum (Artemis) and the corresponding integration vector at a ratio of 9 to 1. For conditional expression of transgenes, cells were cultured in 1 μg/ml doxycycline or 1 μM Shld1. In all experiments, cells were seeded 24 h before induction. To measure the cell proliferation rate, MTS assay was performed according to the manufacturer's instructions (CellTiter 96R Aqueous One Solution Cell Proliferation Assay, Promega).

### Real-time PCR

DNA was extracted using the DNeasy Extraction Kit (Qiagen). Real time PCR was performed using POWR-SYBR Green (Applied Biosystems) on a 7900HT Sequence Detection System (Applied Biosystems). Templates of 10 ng DNA were measured in duplicate. The number of transgene copies was determined by external calibration using the β-actin gene for normalization. Primers used were docking1: 5'-GCAAAGACCCCAACGAGAAG-3', docking2: 5'-TCACGAACTCCAGCAGGACC-3', hACTBf: 5'-GGTATCTCCCTCTGCAGC-3', hACTBr: 5'-CTATGGGCTGAGGTCTGGAT-3'.

### Western blot analysis

Cell pellets were lysed in RIPA buffer (50 mM Tris-HCl pH 7.2, 150 mM NaCl, 0.1% sodium dodecylsulfate, 1% sodium deoxycholate, 1% Triton X-100) supplemented with 0.2% protease inhibitor cocktail (Sigma, P-8340). Insoluble debris were removed by centrifugation at 14,000 g for 10 min at 4°C. The protein concentration of the supernatant was determined using a Bradford assay (Bio-Rad). Equal amounts of proteins were separated on SDS-polyacrylamide gels and transferred to nitrocellulose. Membranes were blocked with blocking reagent (Amersham, RPN 3601). For antigen detection, monoclonal anti-myc tag antibody 9E10, anti-HNF4α (Santa Cruz, C-19), anti-GFP (Clonetech, JL-8 632380), anti-DsRed (Clonetech, 632496) antibodies were employed. Peroxidase-conjugated anti-mouse IgG (Amersham), anti-rabbit IgG (Jackson ImmunoResearch) and anti-goat IgG (Sigma) were used as secondary antibodies. Immunoreactivity was detected by ECL (Amersham Biosciences).

### Plasmids

pDOCKING-Neo (Addgene plasmid 31441) contains the CMV promoter driven ECFP-neomycin fusion protein containing the blue fluorescent protein ECFP (Clonetech). A minimal attP site [15] is placed downstream and in frame of its start codon. In addition, a loxP site was integrated upstream of the start codon. The attB integration vector pINT-PuroDDEYFP (Addgene plasmid 31442) contains a minimal attB site [15] fused in frame to an ATG-less puromycin resistance gene. Downstream of this sequence there is as GOI a CMV promoter driven DD-EYFP fusion protein with the L106P mutant of the human FKBP12 protein (destabilizing domain) [7] linked in frame with the yellow fluorescent protein EYFP (Clonetech). The attB integration vector pINT-PuroDDHNF4α2 (Addgene plasmid 31443) contains as GOI a CMV promoter driven DD-HNF4α2 fusion protein. In the FRT integration vector pcDNA5/FRT/TO-DsRed the DsRed sequence (Clonetech) was introduced into the multiple cloning site of the pcDNA5/FRT/TO vector (Invitrogen) to obtain FR_DsRed2 (Addgene plasmid 31444). The FRT integration vector pcDNA5/FRT/TO-HNF4α2 has been described [3]. All constructs were made using standard molecular procedures. The complete sequence information of all plasmids used can be obtained on request.

## Competing interests

The authors declare that they have no competing interests.

## Authors' contributions

CW designed the project, performed all assays on the HNF4α conditional cells, and drafted the manuscript. OR performed assays on the EYFP/DSRed conditional cells. FS constructed plasmids. MY constructed the docking site. NS performed western blots and transfections. GUR designed and overviewed the project. All authors have read and approved the final version of the manuscript.
